# Associations of genetic polymorphisms of the transporters organic cation transporter 2 (*OCT2*), multidrug and toxin extrusion 1 (*MATE1*), and ATP-binding cassette subfamily C member 2 (*ABCC2*) with platinum-based chemotherapy response and toxicity in non-small cell lung cancer patients

**DOI:** 10.1186/s40880-016-0145-8

**Published:** 2016-09-02

**Authors:** Chen-Yue Qian, Yi Zheng, Ying Wang, Juan Chen, Jun-Yan Liu, Hong-Hao Zhou, Ji-Ye Yin, Zhao-Qian Liu

**Affiliations:** 1 Department of Clinical Pharmacology, Xiangya Hospital, Central South University, Changsha, 410008 Hunan P. R. China; 2 Institute of Clinical Pharmacology, Hunan Key Laboratory of Pharmacogenetics, Central South University, Changsha, 410078 Hunan P. R. China; 3 Hunan Province Cooperation Innovation Center for Molecular Target New Drug Study, Hengyang, 421001 Hunan P. R. China; 4 Key Laboratory of Hunan Province for Traditional Chinese Medicine in Obstetrics & Gynecology Research, The Maternal and Child Health Hospital of Hunan Province, Changsha, 410008 Hunan P. R. China; 5 The Affiliated Cancer Hospital of Xiangya School of Medicine, Central South University, Changsha, 410008 Hunan P. R. China; 6 Xiangya School of Medicine, Central South University, Changsha, 410008 Hunan P. R. China

**Keywords:** OCT2, MATE1, ABCC2, Non-small cell lung cancer, Platinum-based chemotherapy

## Abstract

**Background:**

Platinum-based chemotherapy is the first-line treatment of non-small cell lung cancer (NSCLC); it is therefore important to discover biomarkers that can be used to predict the efficacy and toxicity of this treatment. Four important transporter genes are expressed in the kidney, including organic cation transporter 2 (*OCT2*), multidrug and toxin extrusion 1 (*MATE1*), ATP-binding cassette subfamily B member 1 (*ABCB1*), and ATP-binding cassette subfamily C member 2 (*ABCC2*), and genetic polymorphisms in these genes may alter the efficacy and adverse effects of platinum drugs. This study aimed to evaluate the association of genetic polymorphisms of these transporters with platinum-based chemotherapy response and toxicity in NSCLC patients.

**Methods:**

A total of 403 Chinese NSCLC patients were recruited for this study. All patients were newly diagnosed with NSCLC and received at least two cycles of platinum-based chemotherapy. The tumor response and toxicity were evaluated after two cycles of treatment, and the patients’ genomic DNA was extracted. Seven single-nucleotide polymorphisms in four transporter genes were selected to investigate their associations with platinum-based chemotherapy toxicity and response.

**Results:**

*OCT2* rs316019 was associated with hepatotoxicity (*P* = 0.026) and hematological toxicity (*P* = 0.039), and *MATE1* rs2289669 was associated with hematological toxicity induced by platinum (*P* = 0.016). In addition, *ABCC2* rs717620 was significantly associated with the platinum-based chemotherapy response (*P* = 0.031). *ABCB1* polymorphisms were associated with neither response nor toxicity.

**Conclusion:**

*OCT2* rs316019, *MATE1* rs2289669, and *ABCC2* rs717620 might be potential clinical markers for predicting chemotherapy toxicity and response induced by platinum-based treatment in NSCLC patients.

*Trial registration* Chinese Clinical Trial Registry ChiCTR-RNC-12002892

## Background

Lung cancer is a common cancer and the leading cause of cancer-related deaths in the world [1] and in China [1, 2]. Small cell lung cancer and non–small cell lung cancer (NSCLC) are two major pathologic types of lung cancer. Platinum-based chemotherapy is the first-line treatment for NSCLC; however, drug resistance and toxicity are significant obstacles to successful treatment [3]. Although certain genetic polymorphisms have been reported to be associated with the platinum response and toxicity in NSCLC patients, these results conflict with each other [4–7]. Thus, it is important to discover new biomarkers that can be used to predict platinum-based chemotherapy efficacy and toxicity.

Platinum is mainly eliminated by the proximal tubules in the kidney, and transporters expressed in the kidney play important roles in the distribution and excretion of platinum. Therefore, genetic polymorphisms in these transporters may alter the efficacy and adverse effects of platinum-based chemotherapy [8]. Organic cation transporter 2 (OCT2), encoded by the solute carrier family 22, member 2 (*SLC22A2*) gene, is a major transporter expressed on the basolateral domain of renal tubular cells in the kidney [9]. *OCT2* plays an important role in the uptake of cationic compounds in the kidney, and previous studies have suggested that this transporter plays a potential role in increasing platinum uptake and sensitivity [6, 10]. Multidrug and toxin extrusion 1 (MATE1), encoded by the solute carrier family 47, member 1 (*SLC47A1*) gene, was identified as an H^+^-coupled organic cation exporter [11]. This transporter is mainly expressed on the luminal membranes of the renal urinary duct [12]. *MATE1* is thought to mediate the final step of the renal tubular secretion of platinum [13]. Previous studies showed that platinum was taken up into the renal proximal tubular cells mainly via *OCT2* and secreted into the lumen via *MATE1*; both proteins were thus associated with the disposition of platinum [14]. ATP-binding cassette (ABC) multidrug transporters play an important role in limiting influx and facilitating efflux to prevent the intracellular accumulation of their own substrate compounds [15]. ATP-binding cassette subfamily B member 1 (ABCB1) and ATP-binding cassette subfamily C member 2 (ABCC2) are important members of the ABC transporter family [16]. Studies have suggested that *ABCC2* is involved in the excretion of organic anions, including cisplatin [17], and *ABCC2* has been implicated in platinum resistance and associated toxicity [18].

Our previous investigation showed that gene polymorphisms could be useful clinical markers to assess the platinum response and toxicity in NSCLC patients [19–21]. Because *ABCC2* and *ABCB1* are important members of the ABC family and *OCT2* and *MATE1* are important transporters mainly expressed in the kidney, our investigation focused on these transporters. In this study, we aimed to evaluate the association of seven important single-nucleotide polymorphisms (SNPs) of these transporters with platinum-based chemotherapy response and toxicity in NSCLC patients.

## Methods

### Study subjects

The inclusion criteria for the eligible patients were as follows: (1) newly diagnosed with NSCLC, including adenocarcinoma and squamous cell carcinoma as determined by histology or cytology; (2) received at least two cycles of platinum-based chemotherapy; and (3) tumor response and toxicity could be evaluated after two cycles of treatment. The exclusion criteria included patients who experienced the following: (1) radical or biological therapy before or during the chemotherapy; (2) surgery before or during chemotherapy; (3) current pregnancy or lactation; and (4) an active infection or other concomitant malignancies.

All individuals were recruited from The Affiliated Cancer Hospital or Xiangya Hospital of Central South University (Changsha, Hunan, China) between June 2010 and May 2013. All patients provided written informed consent before they participated in this study. The study protocol was approved by the Ethics Committee of Xiangya School of Medicine, Central South University (Registration number: CTXY-110008-1). The clinical research admission was approved by the Chinese Clinical Trial Registry with the registration number of ChiCTR-RNC-12002892 (http://www.chictr.org/cn/).

### SNP selection, DNA extraction, and genotyping

Seven SNPs in 4 genes (*OCT2*, *ABCB1*, *ABCC2*, and *MATE1*) were selected from the National Center for Biotechnology Information (NCBI) database. All of the SNPs satisfied the following criteria: (1) SNPs that were reported to be associated with lung cancer occurrence or development according to the results of existing research; (2) minor allele frequency (MAF) ≥5% in the Chinese Han population; and (3) tagging SNPs selected by Haploview (version 4.2 Cambridge, MA, USA) using pair-wise tagging with default settings (pair-wise *r*^*2*^ threshold = 0.8).

All blood samples were stored at −20°C until DNA extraction. Within 2 months after the collection of blood samples, genomic DNA was isolated using a Genomic DNA Purification Kit (Promega, Madison, WI, USA) and stored at −20°C before use. Genotyping was conducted using the Sequenom Mass Array Genotype Platform (Sequenom, San Diego, CA, USA).

### Toxicity and response evaluation criteria

The toxicity induced by platinum during chemotherapy was estimated on the basis of the National Cancer Institute Common Toxicity Criteria Version 3.0, in which the extent of toxicity is classified into five grades as follows: grade 1 for mild adverse events; grade 2 for moderate adverse events; grade 3 for severe adverse events; grade 4 for life-threatening or disabling adverse events; and grade 5 for death related to adverse events. We recruited patients who were classified as having grade 0–4 adverse events and divided the patients into two categories. Patients who had grade 0–2 adverse events were regarded as having low toxicity, whereas those with grade 3–4 adverse events were considered as having severe toxicity.

According to the Response Evaluation Criteria in Solid Tumors (RECIST) guidelines (version 1.1), the tumor response was assessed after the first two cycles of chemotherapy by professional clinicians. The responses to therapy were classified into four groups: complete response (CR), partial response (PR), progressive disease (PD), and stable disease (SD). Patients with CR or PR were regarded as being sensitive to platinum, whereas those with SD or PD were considered platinum-resistant.

### Statistical analysis

Patients were dichotomized according to the outcomes of toxicity and response. The allele frequencies of all the SNPs conforming to Hardy–Weinberg equilibrium (HWE) were analyzed with a χ^2^ test (*P* > 0.05). Sex, age, smoking status, tumor histology, clinical stage, and Eastern Cooperative Oncology Group (ECOG) performance status were considered as potential covariates for the logistic regression analysis. The call rate was defined as the percentage of successfully genotyped patients for each SNP. All analyses were performed using PLINK (version 1.07, http://www.pngu.mgh.harvard.edu/purcell/plink/) and SPSS 13.0 (SPSS Inc, Chicago, IL, USA) with three models: (1) additive model for comparing carriers of the minor allele with major allele subjects; (2) dominant model for comparing carriers of the minor allele with the major homozygous subjects; and (3) recessive model for comparing carriers of the major allele with the minor homozygous subjects. Odds ratios (ORs) and their 95% confidence intervals (CIs) were used to assess the associations between outcomes and gene polymorphisms. A value of *P* < 0.05 was considered statistically significant.

## Results

### Patient characteristics

A total of 412 patients who received first-line platinum-based chemotherapy were recruited for this study. After excluding some samples, 403 NSCLC patients were eventually enrolled. Seven SNPs were genotyped in these patients, all of which were conformed in HWE (*P* > 0.05). The basic information on these SNPs and patients is shown in Tables 1 and 2. The median age of the patients was 56 years (range 21–75 years). Severe overall toxicity occurred in 135 (33.5%) patients. Among these, severe hematological toxicity, hepatotoxicity, and gastrointestinal toxicity occurred in 95 (23.6%), 55 (13.6%), and 35 (8.7%) patients, respectively.

**Table 1:** The characteristics of all single-nucleotide polymorphisms (SNPs) involved in the study

Gene	Location	SNP	Alleles	MAF	Call rate (%)	HWE *P* value
*OCT2*	6q25.3	rs316003	C/T	0.78 (T)	96.28	0.903
		rs316019	G/T	0.85 (G)	96.28	0.949
*ABCB1*	7q21.12	rs1045642	C/T	0.38 (T)	98.26	0.969
*ABCC2*	10q24	rs717620	G/A	0.19 (A)	95.53	0.766
		rs2273697	G/A	0.10 (A)	99.75	0.357
		rs3740066	G/A	0.20 (A)	96.77	0.873
*MATE1*	17p11.2	rs2289669	G/A	0.50 (A)	96.03	0.128

*MAF* minor allele frequency, *HWE* Hardy–Weinberg equilibrium, *OCT2* organic cation transporter 2, *ABCB1* ATP-binding cassette subfamily B member 1, *ABCC2* ATP-binding cassette subfamily C member 2, *MATE1* multidrug and toxin extrusion 1

**Table 2:** The clinical characteristics of the 403 non-small cell lung cancer (NSCLC) patients

Variate	Total	Overall toxicity		Hematological toxicity		Hepatotoxicity		Gastrointestinal toxicity		Response^a^	
		Grade 0–2	Grade 3–4	Grade 0–2	Grade 3–4	Grade 0–2	Grade 3–4	Grade 0–2	Grade 3–4	CR + PR	SD + PD
All	403	268 (66.5)	135 (33.5)	308 (76.4)	95 (23.6)	348 (86.4)	55 (13.6)	368 (91.3)	35 (8.7)	115 (29.1)	280 (70.9)
Age (years)											
≤55	176 (43.7)	114 (42.5)	62 (45.9)	139 (45.1)	37 (38.9)	144 (41.4)	32 (58.2)	156 (42.4)	20 (57.1)	54 (47.0)	118 (42.1)
>55	227 (56.3)	154 (57.5)	73 (54.1)	169 (54.9)	58 (61.1)	204 (58.6)	23 (41.8)	212 (57.6)	15 (42.9)	61 (53.0)	162 (57.9)
Smoking status											
Never	170 (42.2)	107 (39.9)	63 (46.7)	131 (42.5)	39 (41.1)	142 (40.8)	28 (50.9)	154 (41.8)	16 (45.7)	46 (40.0)	119 (42.5)
Ever	233 (57.8)	161 (60.1)	72 (53.3)	177 (57.5)	56 (58.9)	206 (59.2)	27 (49.1)	214 (58.2)	19 (54.3)	69 (60.0)	161 (57.5)
Sex											
Male	307 (76.2)	214 (79.9)	93 (68.9)	240 (77.9)	67 (70.5)	269 (77.3)	38 (69.1)	286 (71.7)	21 (60.0)	90 (78.3)	213 (76.1)
Female	96 (23.8)	54 (20.1)	42 (31.1)	68 (22.1)	28 (29.5)	79 (22.7)	17 (30.9)	82 (22.3)	14 (40.0)	25 (21.7)	67 (23.9)
Eastern cooperative oncology group (ECOG) performance status											
0–1	380 (94.3)	255 (95.2)	125 (92.6)	290 (94.2)	90 (94.7)	329 (94.5)	51 (92.7)	348 (94.6)	32 (91.4)	110 (95.7)	263 (93.9)
2	23 (5.7)	13 (4.8)	10 (7.4)	18 (5.8)	5 (5.3)	19 (5.5)	4 (7.3)	20 (5.4)	3 (8.6)	5 (4.3)	17 (6.1)
Histological type											
Adenocarcinoma	217 (53.8)	144 (53.7)	73 (54.1)	180 (58.4)	37 (38.9)	186 (53.4)	31 (56.4)	198 (53.8)	19 (54.3)	68 (59.1)	115 (41.1)
Squamous cell	186 (46.2)	124 (46.3)	62 (45.9)	128 (41.6)	58 (61.1)	162 (46.6)	24 (43.6)	170 (46.2)	16 (45.7)	47 (40.9)	165 (58.9)
TNM stage											
I–II	9 (2.2)	7 (2.6)	2 (1.5)	7 (2.3)	2 (2.1)	9 (2.6)	0 (0.0)	8 (2.2)	1 (2.9)	2 (1.7)	7 (2.5)
III–IV	394 (97.8)	261 (97.4)	133 (98.5)	301 (97.7)	93 (97.9)	339 (97.4)	55 (100.0)	360 (97.8)	34 (97.1)	113 (98.3)	273 (97.5)
Platinum-based drug											
Cisplatin	333 (82.6)	222 (82.8)	111 (82.2)	255 (82.8)	78 (82.1)	284 (81.6)	49 (89.1)	302 (82.1)	31 (88.6)	99 (86.1)	229 (81.8)
Carboplatin	70 (17.4)	46 (17.2)	24 (17.8)	53 (17.2)	17 (17.9)	64 (18.4)	6 (10.9)	66 (17.9)	4 (11.4)	16 (13.9)	51 (18.2)
Chemotherapy regimen											
Platinum-gemcitabine	196 (48.6)	119 (44.4)	77 (57.0)	131 (42.5)	65 (68.4)	168 (48.3)	28 (50.9)	179 (48.6)	17 (48.6)	73 (63.5)	120 (42.8)
Platinum-pemetrexed	148 (36.7)	105 (39.2)	43 (31.9)	127 (41.2)	21 (22.1)	130 (37.4)	18 (32.7)	133 (36.1)	15 (42.8)	27 (23.5)	117 (41.8)
Platinum-paclitaxel	35 (8.7)	27 (10.1)	8 (5.9)	30 (9.8)	5 (5.3)	30 (8.6)	5 (9.1)	33 (9.0)	2 (5.7)	9 (7.8)	26 (9.3)
Platinum-docetaxel	20 (5.0)	14 (5.2)	6 (4.5)	17 (5.5)	3 (3.2)	16 (4.6)	4 (7.3)	19 (5.2)	1 (2.9)	4 (3.5)	15 (5.4)
Platinum-navelbine	4 (1.0)	3 (1.1)	1 (0.7)	3 (1.0)	1 (1.0)	4 (1.1)	0 (0.0)	4 (1.1)	0 (0.0)	2 (1.7)	2 (0.7)

All data are presented as the number of patients with percentage in parentheses

*CR* complete response, *PR* partial response, *PD* progressive disease, *SD* stable disease

^a^The assessment information of 8 patients were lost in the process of sample collection

Tumor response was assessed after the first two cycles of chemotherapy. However, we lost the assessment information of 8 patients in the process of sample collection; the remaining 395 NSCLC patients were finally evaluated, and the basic clinical characteristics of these patients are shown in Table 2. Among them, 115 (29.1%) were regarded as being sensitive to platinum-based chemotherapy, and 280 (70.9%) were regarded as being resistant.

### Associations of *OCT2*, *ABCB1*, *ABCC2,* and *MATE1* polymorphisms with the overall toxicity of platinum-based chemotherapy

As shown in Table 3, *OCT2* rs316019 was significantly associated with the overall toxicity caused by platinum-based chemotherapy in the additive (OR 0.59, 95% CI 0.37–0.93, *P* = 0.024) and dominant (OR 0.59, 95% CI 0.36–0.98, *P* = 0.041) models. Similar results were obtained for *MATE1* rs2289669 (recessive model: OR 1.73, 95% CI 1.06–2.82, *P* = 0.029). *OCT2* rs316019 was also associated with hepatotoxicity in the additive (OR 0.42, 95% CI 0.20–0.90, *P* = 0.026) and dominant models (OR 0.42, 95% CI 0.19–0.93, *P* = 0.033) and with hematological toxicity in the additive model (OR 0.58, 95% CI 0.34–0.97, *P* = 0.039). *MATE1* rs2289669 was significantly associated with hematological toxicity in the recessive model (OR 1.92, 95% CI 1.13–3.25, *P* = 0.016). No SNPs were significantly associated with gastrointestinal toxicity in any of the three models.

**Table 3:** Associations of seven SNPs with platinum-based chemotherapy toxicity in the 403 NSCLC patients

Gene SNP	Genotype	Hepatotoxicity							
		Grade 1–2 [cases (%)]	Grade 3–4 [cases (%)]	Additive model		Dominant model		Recessive model	
				OR (95% CI)	*P*	OR (95% CI)	*P*	OR (95% CI)	*P*
Total		348	55						
*OCT2*									
rs316003	CC	18 (5.2)	1 (1.8)	0.61 (0.34–1.08)	0.089	0.59 (0.31–1.12)	0.109	0.37 (0.05–2.83)	0.336
	CT	120 (34.5)	14 (25.5)						
	TT	298 (85.6)	37 (67.3)						
rs316019	TT	9 (2.6)	0 (0.0)	0.42 (0.20–0.90)	*0.026*	0.42 (0.19–0.93)	*0.033*	0.00	0.998
	GT	87 (25.0)	8 (14.5)						
	GG	239 (68.7)	45 (81.8)						
*ABCB1*									
rs1045642	TT	51 (14.7)	6 (10.9)	0.81 (0.52–1.26)	0.354	0.75 (0.42–1.35)	0.337	0.81 (0.32–2.01)	0.643
	CT	165 (47.4)	24 (43.6)						
	CC	126 (36.2)	24 (43.6)						
*ABCC2*									
rs717620	AA	10 (2.9)	2 (3.6)	0.93 (0.54–1.59)	0.782	0.89 (0.48–1.66)	0.712	1.11 (0.23–5.44)	0.895
	AG	104 (29.9)	15 (27.3)						
	GG	216 (62.1)	38 (69.1)						
rs2273697	AA	2 (0.6)	0 (0.0)	1.15 (0.58–2.28)	0.682	1.20 (0.59–2.44)	0.608	0.00	0.999
	AG	60 (17.2)	12 (21.8)						
	GG	285 (81.9)	43 (78.2)						
rs3740066	AA	12 (3.4)	2 (3.6)	0.69 (0.38–1.23)	0.206	0.60 (0.31–1.17)	0.134	1.04 (0.22–4.92)	0.958
	AG	113 (32.5)	11 (20.0)						
	GG	213 (61.2)	39 (70.9)						
*MATE1*									
rs2289669	AA	79 (22.7)	10 (18.2)	0.86 (0.56–1.33)	0.500	0.92 (0.46–1.82)	0.804	0.73 (0.35–1.53)	0.402
	AG	175 (50.3)	30 (54.5)						
	GG	80 (23.0)	13 (23.6)						

Gene SNP	Genotype	Gastrointestinal toxicity							
		Grade 1–2 [cases (%)]	Grade 3–4 [cases (%)]c	Additive model		Dominant model		Recessive model	
				OR (95% CI)	*P*	OR (95% CI)	*P*	OR (95% CI)	*P*
Total		368	35						
*OCT2*									
rs316003	CC	17 (4.6)	2 (5.7)	1.27 (0.72–2.25)	0.412	1.40 (0.69–2.85)	0.357	1.14 (0.25–5.23)	0.870
	CT	120 (32.6)	14 (40.0)						
	TT	217 (59.0)	18 (51.4)						
rs316019	TT	8 (2.2)	1 (2.9)	0.49 (0.20–1.17)	0.106	0.39 (0.15–1.05)	0.062	1.16 (0.14–9.74)	0.893
	GT	91 (24.7)	4 (11.4)						
	GG	254 (69.0)	30 (85.7)						
*ABCB1*									
rs1045642	TT	52 (14.1)	5 (14.3)	0.71 (0.41–1.22)	0.217	0.54 (0.26–1.10)	0.091	0.96 (0.35–2.63)	0.941
	CT	177 (48.1)	12 (34.3)						
	CC	133 (36.1)	17 (48.6)						
*ABCC2*									
rs717620	AA	11 (3.0)	1 (2.9)	0.79 (0.38–1.61)	0.510	0.73 (0.32–1.63)	0.438	1.09 (0.13–8.81)	0.939
	AG	111 (30.2)	8 (22.9)						
	GG	231 (62.8)	23 (65.7)						
rs2273697	AA	2 (0.5)	0 (0.0)	1.94 (0.93–4.04)	0.078	2.10 (0.97–4.53)	0.060	0.00	0.999
	AG	61 (16.6)	11 (31.4)						
	GG	304 (82.6)	24 (68.6)						
rs3740066	AA	12 (3.3)	2 (5.7)	0.91 (0.47–1.79)	0.795	0.76 (0.35–1.67)	0.500	2.25 (0.47–10.75)	0.309
	AG	116 (31.5)	8 (22.9)						
	GG	229 (62.2)	23 (65.7)						
*MATE1*									
rs2289669	AA	79 (21.5)	10 (28.6)	1.35 (0.80–2.28)	0.254	1.55 (0.62–3.89)	0.350	1.44 (0.66–3.16)	0.363
	AG	187 (50.8)	18 (51.4)						
	GG	87 (23.6)	6 (17.1)						

Gene SNP	Genotype	Hematological toxicity							
		Grade 1–2 [cases (%)]	Grade 3–4 [cases (%)]	Additive model		Dominant model		Recessive model	
				OR (95% CI)	*P*	OR (95% CI)	*P*	OR (95% CI)	*P*
Total		308	95						
*OCT2*									
rs316003	CC	17 (5.5)	2 (2.1)	0.75 (0.49–1.15)	0.190	0.78 (0.47–1.28)	0.324	0.37 (0.08–1.67)	0.196
	CT	103 (33.4)	31 (32.6)						
	TT	176 (57.1)	59 (62.1)						
rs316019	TT	9 (2.9)	0 (0.0)	0.58 (0.34–0.97)	*0.039*	0.61 (0.34–1.07)	0.085	0.00	0.998
	GT	75 (24.4)	20 (21.1)						
	GG	209 (67.9)	75 (78.9)						
*ABCB1*									
rs1045642	TT	44 (14.3)	13 (13.7)	0.92 (0.65–1.31)	0.648	0.87 (0.53–1.41)	0.560	0.97 (0.49–1.93)	0.936
	CT	147 (47.7)	42 (44.2)						
	CC	111 (36.0)	39 (41.1)						
*ABCC2*									
rs717620	AA	8 (2.6)	4 (4.2)	1.15 (0.74–1.78)	0.546	1.09 (0.66–1.81)	0.736	1.88 (0.53–6.72)	0.330
	AG	91 (29.5)	28 (29.5)						
	GG	196 (63.6)	58 (61.1)						
rs2273697	AA	1 (0.3)	1 (1.1)	1.13 (0.64–1.98)	0.679	1.10 (0.60–1.99)	0.759	2.46 (0.15–40.00)	0.528
	AG	54 (17.5)	18 (18.9)						
	GG	252 (81.8)	76 (80.0)						
rs3740066	AA	12 (3.9)	2 (2.1)	0.89 (0.58–1.38)	0.600	0.94 (0.57–1.55)	0.806	0.49 (0.11–2.27)	0.361
	AG	94 (30.5)	30 (31.6)						
	GG	192 (62.3)	60 (63.2)						
*MATE1*									
rs2289669	AA	58 (18.8)	31 (32.6)	1.30 (0.92–1.83)	0.141	0.99 (0.57–1.72)	0.973	1.92 (1.13–3.25)	*0.016*
	AG	164 (53.2)	41 (43.2)						
	GG	70 (22.7)	23 (24.2)						

Gene SNP	Genotype	Overall toxicity							
		Grade 1–2 [cases (%)]	Grade 3–4 [cases (%)]	Additive model		Dominant model		Recessive model	
				OR (95% CI)	*P*	OR (95% CI)	*P*	OR (95% CI)	*P*
Total		268	135						
*OCT2*									
rs316003	CC	14 (5.2)	5 (3.7)	0.85 (0.59–1.23)	0.400	0.86 (0.56–1.33)	0.502	0.66 (0.23–1.90)	0.446
	CT	91 (34.0)	43 (31.9)						
	TT	154 (57.5)	81 (60.0)						
rs316019	TT	8 (3.0)	1 (0.7)	0.59 (0.37–0.93)	*0.024*	0.59 (0.36–0.98)	*0.041*	0.21 (0.03–1.75)	0.150
	GT	68 (25.4)	27 (20.0)						
	GG	179 (66.8)	105 (77.8)						
*ABCB1*									
rs1045642	TT	39 (14.6)	18 (13.3)	0.91 (0.67–1.24)	0.539	0.89 (0.58–1.38)	0.604	0.86 (0.47–1.58)	0.632
	CT	126 (47.0)	63 (46.7)						
	CC	98 (36.6)	52 (38.5)						
*ABCC2*									
rs717620	AA	7 (2.6)	5 (3.7)	1.04 (0.70–1.54)	0.838	1.00 (0.63–1.56)	0.985	1.52 (0.47–4.91)	0.489
	AG	80 (29.9)	39 (28.9)						
	GG	169 (63.1)	85 (63.0)						
rs2273697	AA	1 (0.4)	1 (0.7)	1.49 (0.90–2.54)	0.118	1.50 (0.89–2.53)	0.128	2.30 (0.14–37.24)	0.557
	AG	42 (15.7)	30 (22.2)						
	GG	224 (83.6)	104 (77.0)						
rs3740066	AA	11 (4.1)	3 (2.2)	0.86 (0.58–1.27)	0.446	0.88 (0.56–1.37)	0.573	0.58 (0.16–2.12)	0.409
	AG	83 (31.0)	41 (30.4)						
	GG	165 (61.6)	87 (64.4)						
*MATE1*									
rs2289669	AA	50 (18.7)	39 (28.9)	1.30 (1.00–1.86)	0.052	1.28 (0.77–2.13)	0.335	1.73 (1.06–2.82)	*0.029*
	AG	140 (52.2)	65 (48.1)						
	GG	65 (24.3)	28 (20.7)						

*OCT2* organic cation transporter 2, *ABCB1* ATP-binding cassette subfamily B member 1, *ABCC2* ATP-binding cassette subfamily C member 2, *MATE1* multidrug and toxin extrusion 1

**Fig. 1:**
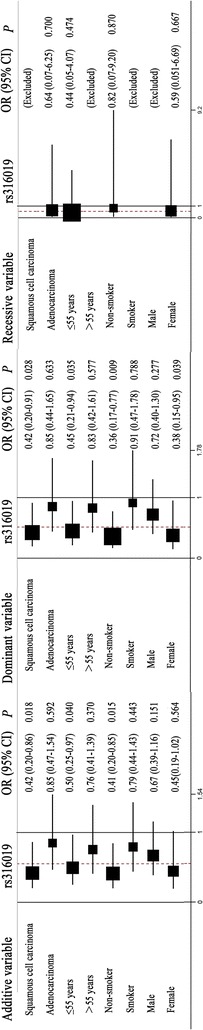
Stratification analyses of the association of the organic cation transporter 2 (*OCT2*) rs316019 polymorphisms with the overall toxicity of platinum-based chemotherapy in non-small cell lung cancer (*NSCLC*) patients using the additive, dominant, and recessive models. Each *box* and *horizontal* *line* represents the values of the odds ratio (OR) and 95% confidence interval (95% CI)

**Fig. 2:**
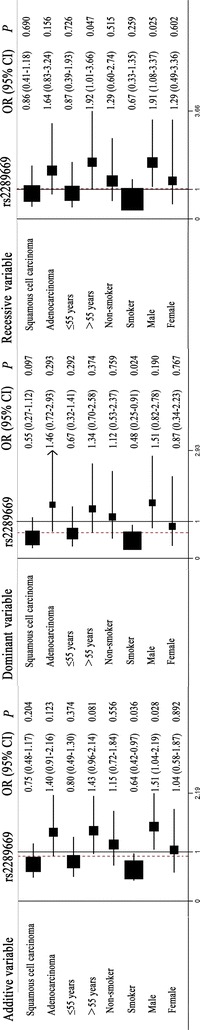
Stratification analyses of the association of the multidrug and toxin extrusion 1 (*MATE1*) rs2289669 polymorphisms with the overall toxicity of platinum-based chemotherapy in NSCLC patients using the additive, dominant, and recessive models. Each *box* and *horizontal line* represents the values of the OR and 95% CI. The *arrow* indicates that the region of the OR was wider than the raw data set according to the computer analysis

Stratification analyses were performed to further examine overall toxicity (Figs. 1, 2). The patients were stratified according to four clinical characteristics: squamous cell carcinoma or adenocarcinoma; ≤55 or >55 years of age; non-smoker or smoker; and male or female. *OCT2* rs316019 was significantly associated with squamous carcinoma (additive model: OR 0.42, 95% CI 0.20–0.86, *P* = 0.018; dominant model: OR 0.42, 95% CI 0.20–0.91, *P* = 0.028), patients ≤55 years of age (additive model: OR 0.50, 95% CI 0.25–0.97, *P* = 0.040; dominant model: OR 0.45, 95% CI 0.21–0.94, *P* = 0.035), being a non-smoker (additive model: OR 0.41, 95% CI 0.20–0.85, *P* = 0.015; dominant model: OR 0.36, 95% CI 0.17–0.77, *P* = 0.009), and female patients (dominant model: OR 0.38, 95% CI 0.15–0.95, *P* = 0.039). In contrast, *MATE1* rs2289669 was significantly associated with overall toxicity for patients >55 years of age (recessive model: OR 1.92, 95% CI 1.01–3.66, *P* = 0.047), being a smoker (additive model: OR 0.64, 95% CI 0.42–0.97, *P* = 0.036; dominant model: OR 0.48, 95% CI 0.25–0.91, *P* = 0.024), and male patients (additive model: OR 1.51, 95% CI 1.04–2.19, *P* = 0.028; recessive model: OR 1.91, 95% CI 1.08–3.37, *P* = 0.025).

Taken together, these results indicate that G allele carriers of *OCT2* rs316019 polymorphisms have better tolerance to hematological toxicity and hepatotoxicity, whereas A allele carriers of rs2289669 polymorphisms have poor tolerance to hematological toxicity caused by platinum-based chemotherapy.

### Associations of *OCT2*, *ABCB1*, *ABCC2,* and *MATE1* polymorphisms with platinum-based chemotherapy responses

The platinum-based chemotherapy responses of 395 NSCLC patients are summarized in Table 4. There was a significant association of *ABCC2* rs717620 with chemotherapy response in the additive (OR 0.64, 95% CI 0.43–0.96, *P* = 0.006) and dominant models (OR 0.54, 95% CI 0.34–0.86, *P* = 0.010). However, no significant association was found between other SNPs and chemotherapy response.

**Table 4:** Associations of seven SNPs with platinum-based chemotherapy response in 395 NSCLC patients

Gene SNP	Genotype	Response							
		Sensitive [cases (%)]	Resistant [cases (%)]	Additive model		Dominant model		Recessive model	
				OR (95% CI)	*P*	OR (95% CI)	*P*	OR (95% CI)	*P*
Total		115	280						
*OCT2*									
rs316003	CC	5 (4.3)	13 (4.6)	1.00 (0.68–1.47)	0.992	1.01 (0.63–1.60)	0.984	0.96 (0.33–2.81)	0.939
	CT	38 (33.0)	93 (33.2)						
	TT	65 (56.5)	166 (59.3)						
rs316019	TT	2 (1.7)	7 (2.5)	1.08 (0.69–1.69)	0.741	1.05 (0.63–1.75)	0.840	1.51 (0.30–7.51)	0.616
	GT	27 (23.5)	68 (24.3)						
	GG	79 (68.7)	197 (70.4)						
*ABCB1*									
rs1045642	TT	10 (8.7)	45 (16.1)	1.18 (0.85–1.65)	0.323	1.05 (0.66–1.66)	0.834	1.84 (0.89–3.83)	0.102
	CT	57 (49.6)	129 (46.1)						
	CC	45 (39.1)	103 (36.8)						
*ABCC2*									
rs717620	AA	3 (2.6)	9 (3.2)	0.64 (0.43–0.96)	*0.031*	0.54 (0.34–0.86)	*0.010*	1.22 (0.32–4.68)	0.772
	AG	46 (40.0)	72 (25.7)						
	GG	62 (53.9)	186 (66.4)						
rs2273697	AA	1 (0.9)	1 (0.4)	1.29 (0.73–2.28)	0.391	1.35 (0.74–2.47)	0.328	0.59 (0.04–9.55)	0.709
	AG	16 (13.9)	52 (18.6)						
	GG	97 (84.3)	227 (81.1)						
rs3740066	AA	4 (3.5)	10 (3.6)	0.80 (0.54–1.19)	0.271	0.72 (0.45–1.14)	0.162	1.18 (0.36–3.90)	0.790
	AG	42 (36.5)	81 (28.9)						
	GG	65 (56.5)	181 (64.6)						
*MATE1*									
rs2289669	AA	20 (17.4)	65 (23.2)	1.30 (0.93–1.81)	0.126	1.32 (0.79–2.21)	0.293	1.51 (0.86–2.66)	0.155
	AG	58 (50.4)	140 (50.0)						
	GG	30 (26.1)	62 (22.1)						

*OCT2* organic cation transporter 2, *ABCB1* ATP-binding cassette subfamily B member 1, *ABCC2* ATP-binding cassette subfamily C member 2, *MATE1* multidrug and toxin extrusion 1

**Fig. 3:**
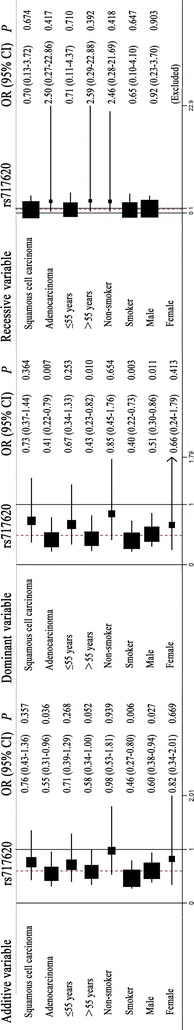
Stratification analyses of the association of the ATP-binding cassette subfamily C member 2 (*ABCC2*) rs717620 polymorphisms and the efficacy of platinum-based chemotherapy in NSCLC patients using the additive, dominant, and recessive models. Each *box* and *horizontal line* represents the values of OR and 95% CI. The *arrow* indicates that the region of the OR was wider than the raw data set according to the computer analysis

Additional stratification analyses are shown in Fig. 3. *ABCC2* rs717620 was significantly associated with chemotherapy response for adenocarcinoma patients (additive model: OR 0.55, 95% CI 0.31–0.96, *P* = 0.036; dominant model: OR 0.41, 95% CI 0.22–0.79, *P* = 0.007), patients >55 years of age (dominant model: OR 0.43, 95% CI 0.23–0.82, *P* = 0.010), smoker patients (additive model: OR 0.46, 95% CI 0.27–0.80, *P* = 0.006; dominant model: OR 0.40, 95% CI 0.22–0.73, *P* = 0.003), and male patients (additive model: OR 0.60, 95% CI 0.38–0.94, *P* = 0.027; dominant model: OR 0.51, 95% CI 0.30–0.86, *P* = 0.011).

Therefore, we concluded that A allele carriers of *ABCC2* rs717620 polymorphisms have a better response to platinum-based chemotherapy, especially those who are >55 years of age, smokers, or male.

## Discussion

In this study, we investigated whether polymorphisms in transporter genes (*OCT2*, *ABCB1*, *ABCC2*, and *MATE1*) were associated with toxicity and the response to platinum-based chemotherapy in 403 NSCLC patients. We evaluated the associations of these gene polymorphisms with gastrointestinal toxicity, hematological toxicity, hepatotoxicity, and overall toxicity and performed a stratification analysis of overall toxicity to extract additional information. Our results showed that *OCT2* rs316019 was associated with hepatotoxicity and hematological toxicity, whereas *MATE1* rs2289669 was associated with hematological toxicity induced by platinum. However, there was no statistical association between either *OCT2* rs316019 or *MATE1* rs2289669 polymorphisms and the platinum-based chemotherapy response in these patients. We found that only *ABCC2* rs717620 may be related to chemotherapy response in NSCLC patients.

Our results showed that *OCT2* rs316019 was associated with platinum-induced hematological toxicity, hepatotoxicity, and overall toxicity. G allele carriers had better tolerance to hematological toxicity and hepatotoxicity after platinum-based chemotherapy. Moreover, NSCLC patients ≤55 years old, non-smokers, or those diagnosed with squamous cell carcinoma carrying the G allele of *OCT2* rs316019 presented a lower risk of overall severe toxicity than their counterparts. This polymorphism was also associated with chemotherapy toxicity for female patients in the present study. All these results indicated that *OCT2* rs316019 was associated with reduced cisplatin-induced hematological toxicity and hepatotoxicity in Chinese NSCLC patients.

Numerous variations of the *MATE1* gene have been determined to affect the clinical response to cationic drugs. It has also been reported that *MATE1* transporter activity was reduced by rs2289669 [22]. Indeed, our results demonstrated that *MATE1* rs2289669 was associated with hematological toxicity and overall toxicity during platinum-based chemotherapy. A allele carriers had poor tolerance to hematological toxicity and overall toxicity in the recessive model. Furthermore, NSCLC patients >55 years old in the recessive model, patients who were smokers in the additive and dominant models, and male patients in the additive and recessive models showed associations with overall toxicity in platinum-based chemotherapy.

*ABCC2* is an efflux transporter expressed in the bile canalicular membrane, and this protein plays a primary role in the metabolism of many chemotherapeutic agents [23]. *ABCC2* expression is present in many tumor tissues and may lead to multidrug resistance [18]. In 1999, it was found that *ABCC2* modulated cisplatin resistance through glutathione transport; a mutation in *ABCC2* could thus highly influence the sensitivity to cisplatin [24]. In the present study, *ABCC2* rs717620 was demonstrated to be associated with the response to platinum-based chemotherapy. Patients who carried the A allele of the rs2289669 polymorphism had better tolerance to hematological toxicity. Furthermore, we found that the *ABCC2* rs717620 polymorphism increased the sensitivity to platinum in patients diagnosed with adenocarcinoma, smoker patients, and male patients.

It should be noted that some previous studies reported results that are different from our present investigation. For example, one study showed no association between *MATE1* rs2289669 and platinum toxicity. *ABCC2* rs717620 had also been found to be moderately associated with a poor response to chemotherapy, which was opposite to our results [15]. Although the detailed reasons for these conflicting results remain unknown, we speculate that these inconsistencies maybe due to small sample sizes and different ethnic populations. In addition, when we calculated the *P* values for all the SNPs associated with toxicity and response with a false detection rate correction, no SNPs had a statistically significant association. Therefore, to obtain a more solid conclusion, independent replication studies with larger sample sizes should be performed in the future to validate our findings.

## Conclusions

Our findings showed that, among Chinese NSCLC patients, carriers of the G allele of *OCT2* rs316019 had better toxicity tolerance, whereas carriers of the A allele of *MATE1* rs2289669 had poor toxicity tolerance, and carriers of the A allele of *ABCC2* rs717620 responded better to platinum-based chemotherapy. These SNPs are potential clinical markers for predicting the response to platinum-based chemotherapy in NSCLC patients. However, further studies with a larger sample size are warranted to validate these findings.
